# Comparative efficacy of exercise modalities for mild cognitive impairment in older adults: A network meta-analysis

**DOI:** 10.1016/j.isci.2026.117561

**Published:** 2026-10-07

**Authors:** Yixuan Wu, Yin Wu

**Affiliations:** 1 The First Affiliated Hospital of Soochow University, Suzhou, China; 2 School of Nursing, Soochow University, Suzhou, China

**Keywords:** MCI, mild cognitive impairment, older people

## Abstract

Mild cognitive impairment (MCI) represents a critical window for dementia prevention, yet the comparative efficacy of diverse exercise modalities remains unclear. We conducted a frequentist network meta-analysis of 16 randomized controlled trials to identify the optimal exercise intervention for improving global cognition in older adults with MCI. Combined physical-cognitive training ranked first with a 68.0% probability of being the most effective intervention, followed by Tai Chi (15.9%), while single-mode exercises and control groups showed substantially lower benefits. Interventions requiring simultaneous physical exertion and neural engagement confer superior cognitive benefits. This evidence supports tailored dual-component exercise regimens as a robust non-pharmacological strategy to mitigate cognitive decline in aging populations.

## Highlights

• Network meta-analysis evaluated exercise modalities for MCI older adults
• Combined physical-cognitive exercise ranked best for improving cognition
• Tai Chi and coordination training also showed superior cognitive benefits
• Dual-component exercise is a robust strategy to mitigate cognitive decline

## Teaser

Kinesiology; Physiological state

## Introduction

Mild cognitive impairment (MCI) represents a critical transitional stage between normal cognitive aging and early dementia, particularly Alzheimer’s disease (AD).^1^ With the global aging population expanding rapidly, the prevalence of MCI is surging, imposing a substantial burden on healthcare systems.^2^ Given the current lack of effective disease-modifying pharmacological treatments for dementia, identifying effective preventive strategies during the MCI stage has become a global public health priority.^3^ Among non-pharmacological interventions, physical exercise has emerged as a cornerstone therapy, demonstrating robust potential to preserve global cognition and mitigate neural degeneration.^4^^,^^5^

To date, diverse exercise modalities have been investigated for their cognitive benefits in older adults with MCI. These include conventional aerobic exercise (e.g., walking, cycling), resistance training, multicomponent exercise, coordination training, and traditional mind-body exercises such as Tai Chi.^6^^,^^7^ Recent trends have also highlighted the efficacy of “combined training” or “dual-task” interventions, which integrate physical exertion with concurrent cognitive challenges.^8^ The prevailing hypothesis suggests that physical movement enhances cerebral blood flow and the upregulation of neurotrophic factors (e.g., BDNF), while cognitive engagement facilitates neural network remodeling and synaptic plasticity.^9^

Despite the wealth of evidence supporting the general cognitive benefits of exercise, a critical knowledge gap remains. Previous pairwise meta-analyses have largely focused on comparing a single exercise type against a control group, or broadly categorizing interventions into “mind-body” versus “conventional” exercises.^10^^,^^11^ Consequently, the comparative efficacy of these diverse and specific modalities remains poorly defined. Without direct head-to-head comparisons across all available interventions, clinicians, and physical therapists face a “black box” when attempting to prescribe the most effective exercise regimen. It is still unclear whether demanding neural-motor coordination (e.g., Tai Chi) or concurrent cognitive-physical loads (e.g., combined exercise) provides superior cognitive benefits over simple aerobic activities.

To address this critical gap and facilitate precise clinical decision-making, we conducted a systematic review and frequentist network meta-analysis (NMA) of randomized controlled trials (RCTs). Unlike traditional meta-analyses, NMA allows for the simultaneous synthesis of both direct and indirect evidence across a network of interventions.^12^ The primary objective of this study is to evaluate the relative effectiveness of different specific exercise modalities (aerobic, multicomponent, coordination, Tai Chi, and combined training) and to determine the optimal exercise prescription for improving global cognition in older adults with MCI.

## Results

### Study selection

The initial database search yielded a total of 6,737 records. After removing duplicates and screening titles and abstracts, 5,872 studies were sought for full-text recovery. Following a rigorous full-text evaluation based on the inclusion and exclusion criteria, 16 RCTs were ultimately included in this NMA (Figure 1).

**Figure 1:**
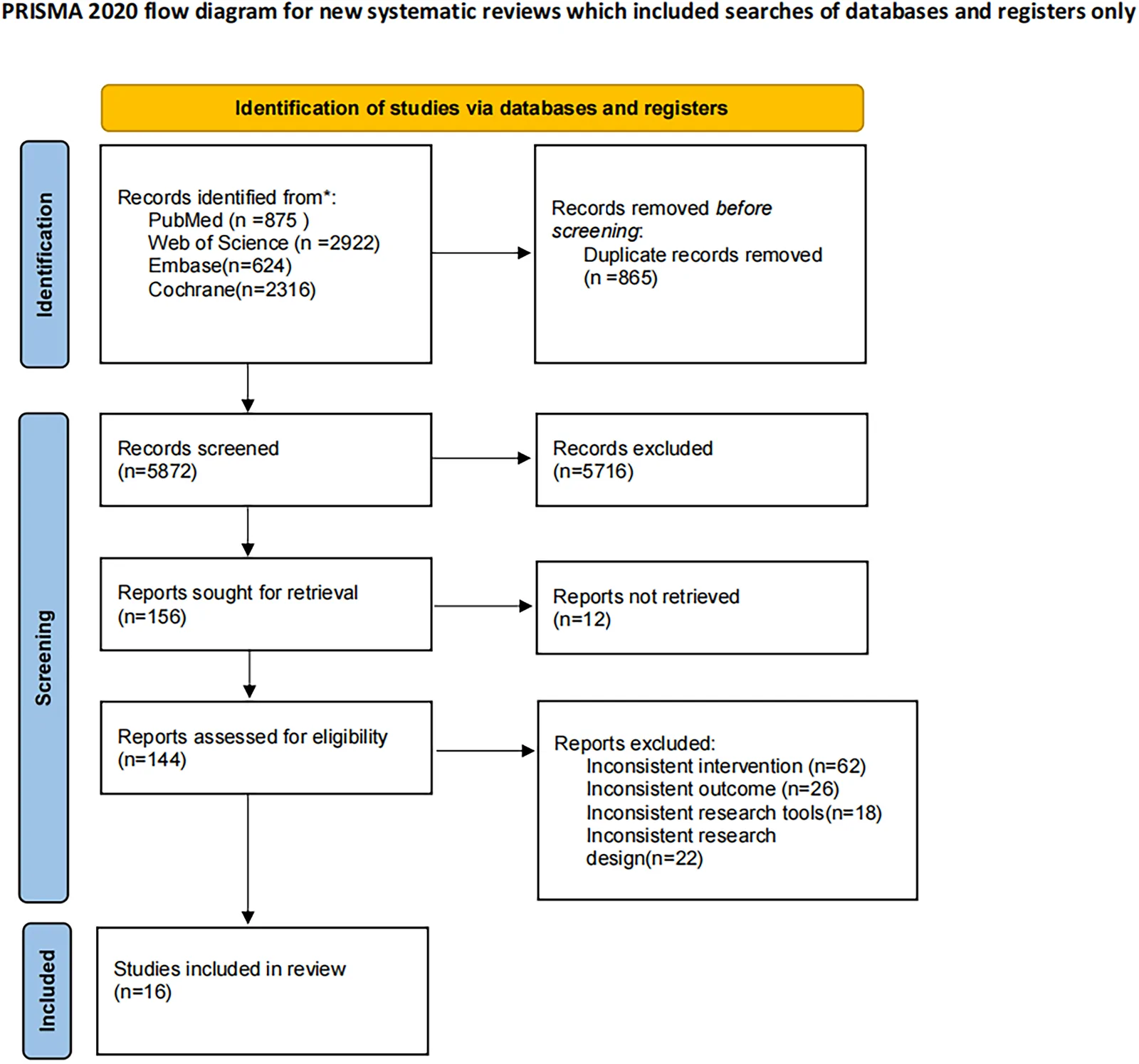
PRISMA flow diagram of study selection

### Characteristics of included trials

The 16 included RCTs involved a total of 1,208 participants with mild cognitive impairment (MCI). The sample sizes of individual studies ranged from 24 to 136. The mean age of participants across all trials was over 65 years. Geographically, the studies were conducted in China (*n* = 7), South Korea (*n* = 1), Pakistan (*n* = 3), Brazil (*n* = 2), Turkey (*n* = 1), Poland (*n* = 1), and Japan (*n* = 1). The intervention duration varied from 12 to 24 weeks, with a frequency of 2–3 sessions per week. Global cognitive function was measured using the Montreal Cognitive Assessment (MoCA) or the Mini-Mental State Examination (MMSE). Detailed baseline characteristics are summarized in Table 1.

**Table 1:** Characteristics of included randomized controlled trials

Study(Year)	Country	Sample size(total/F)	Age(mean ± SD)	Specific diagnostic criteria for MCI	Interventions(arms)	Control group	Duration(weeks)	Frequency/time	Outcome measures
Amjad et al.^13^	Pakistan	33 (47.5)	67.85 ± 5.61	MMSE, MoCA<25	aerobic vs. active control	stretching	12	3 times/wk, 40–50 min	MMSE
Bademli et al.^14^	Turkey	60 (41.7%)	71.9 ± 5.1	NR	multicomponent vs. passive control	daily routine	12	2 times/wk, 60 min	MMSE
Bae et al.^15^	South Korea	56 (51.8%)	76.5 ± 4.5	MMSE≤24	multicomponent vs. active control	health education	12	2 times/wk, 60 min	MMSE
Chang et al.^16^	China	109 (100%)	71.5 ± 5.2	MoCA<26	aerobic vs. passive control	daily routine	12	3 times/wk, 45 min	MoCA
DeSá et al.^17^	Brazil	40 (77.8%)	68.5 ± 4.2	CDR:0.5–1.0	multicomponent vs. combined vs. coordination	motor tasks	24	3 times/wk, 60 min	MoCA
Khattak et al.^18^	Pakistan	59 (NR)	69.2 ± 4.8	MMSE:18∼23	aerobic vs. active control	gentle active range of motion exercises, stretching	12	3 times/wk, 45 min	MoCA
Khattak et al.^19^	Pakistan	51 (49.0%)	70.1 ± 5.3	MMSE:18∼23	multicomponent vs. aerobic	treadmill walk	12	3 times/wk, 45 min	MoCA
Langoni et al.^20^	Brazil	52 (23.1)	74.2 ± 6.1	Pfeffer’s functional assessment questionnaire	multicomponent vs. passive control	daily routine	24	2 times/wk, 60 min	MMSE
Li et al.^21^	China	84 (60.7%)	66.8 ± 3.9	MoCA<25	multicomponent vs. active control	regular community health instruction	12	3 times/wk, 60 min	MoCA
Song et al.^22^	China	95 (25%)	68.4 ± 4.7	MoCA:19∼26	aerobic vs. active control	health education	12	3 times/wk, 50 min	MoCA
Suzuki et al.^23^	Japan	92 (49%)	75.3 ± 4.1	NR	multicomponent vs. active control	health education	24	2 times/wk, 90 min	MoCA
Wang et al.^24^	China	60 (NR)	72.1 ± 5.6	MoCA<26	aerobic vs. passive control	daily routine	12	3 times/wk, 45 min	MoCA
Wang et al.^25^	China	61 (71.2%)	69.8 ± 4.2	MoCA<26	aerobic vs. passive control	daily routine	12	3 times/wk, 45 min	MoCA
Wei et al.^26^	China	60 (33.3%)	70.5 ± 5.3	MMSE≤26	multicomponent vs. passive control	daily routine	24	3 times/wk, 60 min	MMSE
Yu et al.^27^	China	28 (NR)	73.4 ± 4.8	MoCA-HK	Tai-Chi vs. multicomponent vs. passive control	daily routine	12	2 times/wk, 60min	MoCA
Jurakic et al.^28^	Poland	28 (100%)	70.4 ± 3.9	MoCA:19∼25	Huber training	pilates training	24	3 times/wk, 60 min	MoCA

### Risk of bias in included studies

Methodological quality was assessed using the Cochrane RoB 2.0 tool (Figure 2). Overall, the majority of the included studies were graded as having a “low risk” of bias. In the “randomization process” domain, several studies (*n* = 6) provided sufficient detail on sequence generation and allocation concealment. Bias due to “deviations from intended interventions” and “missing outcome data” was low across almost all trials. While blinding of participants is inherently challenging in exercise interventions, the bias in “measurement of the outcome” was generally low as cognitive assessments were conducted using standardized tools.

**Figure 2:**
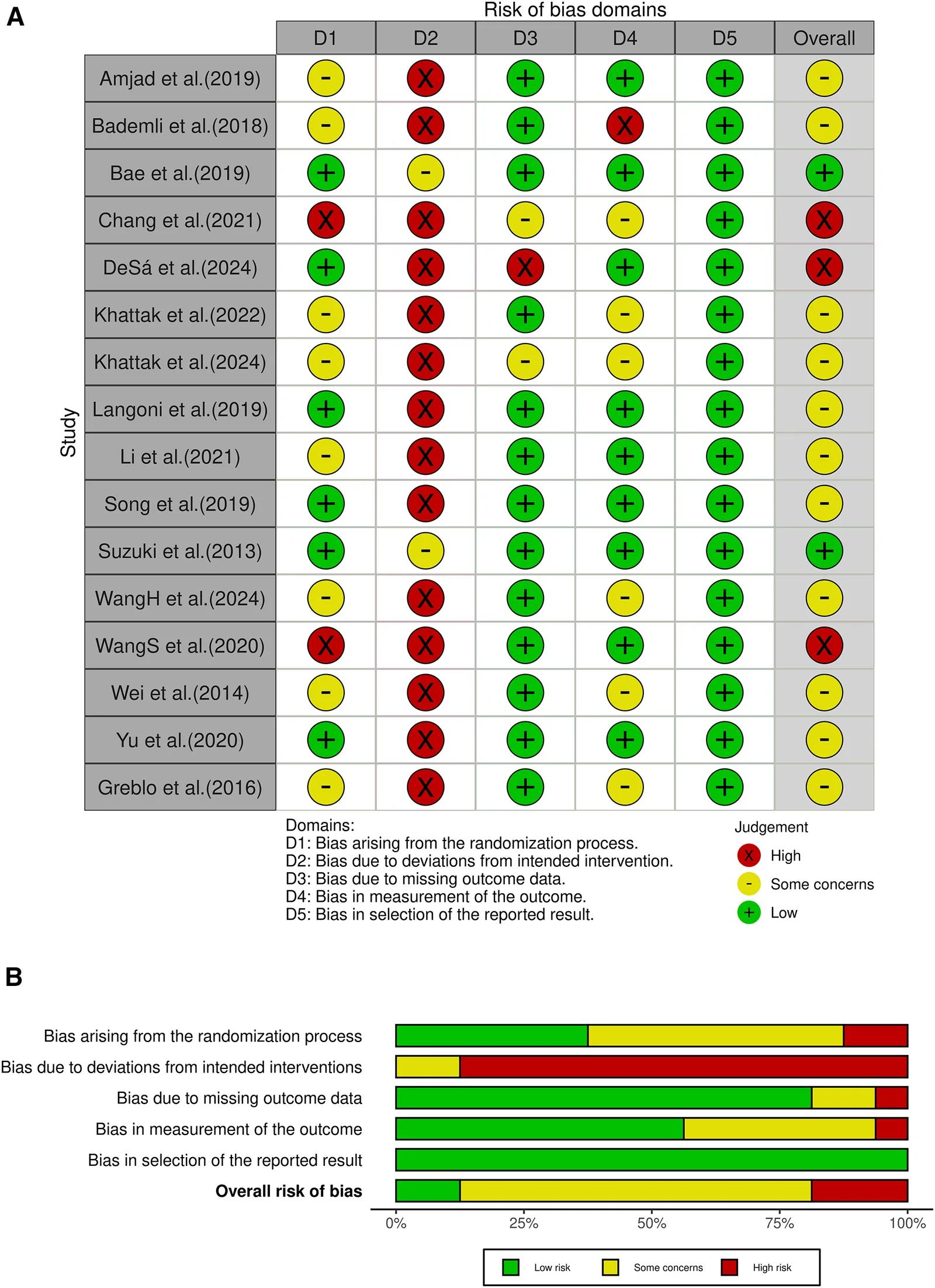
Risk of bias assessment for included randomized controlled trials

### Effects of interventions on global cognitive function

The evidence network for the primary outcome (global cognition) is illustrated in Figure 3. All 16 studies were well-connected in a single network, allowing for both direct and indirect comparisons.

**Figure 3:**
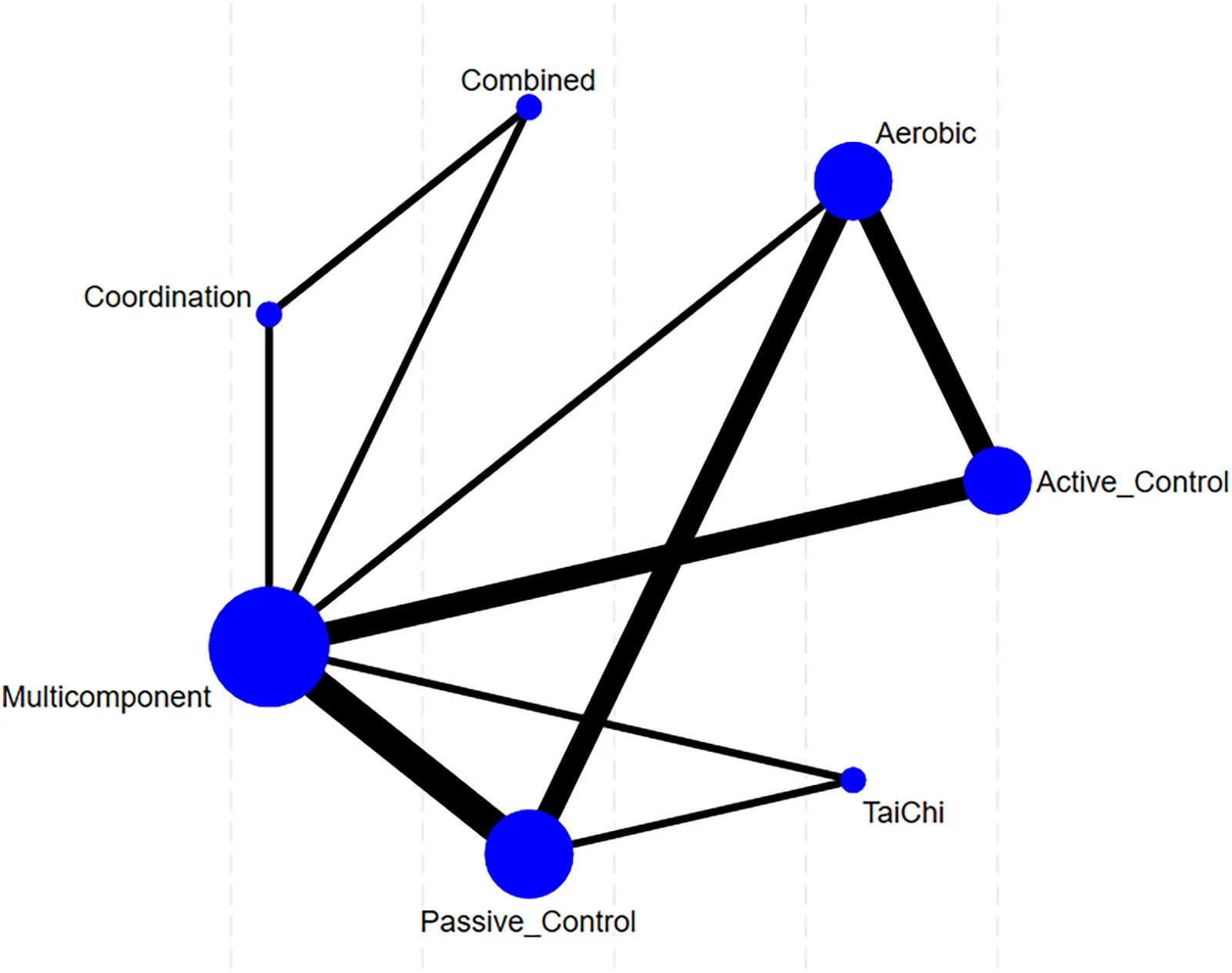
Evidence network plot of diverse exercise modalities for global cognitive function

The network forest plot (Figure 4) presents the comparative efficacy of various exercise modalities against the active control group. Compared with the active control, combined exercise showed the most significant improvement in cognitive scores (SMD = 3.78; 95% CI: 0.89 to 6.67). Significant positive effects were also observed for multicomponent exercise (SMD = 1.71; 95% CI: 0.50 to 2.92) and aerobic exercise (SMD = 1.47; 95% CI: 0.25 to 2.69). Although Tai Chi (SMD = 2.22; 95% CI: −0.37 to 4.81) and coordination training (SMD = 2.50; 95% CI: −0.38 to 5.39) showed positive trends, their 95% confidence intervals (CIs) crossed the line of no effect, likely due to smaller sample sizes in these specific arms.

**Figure 4:**
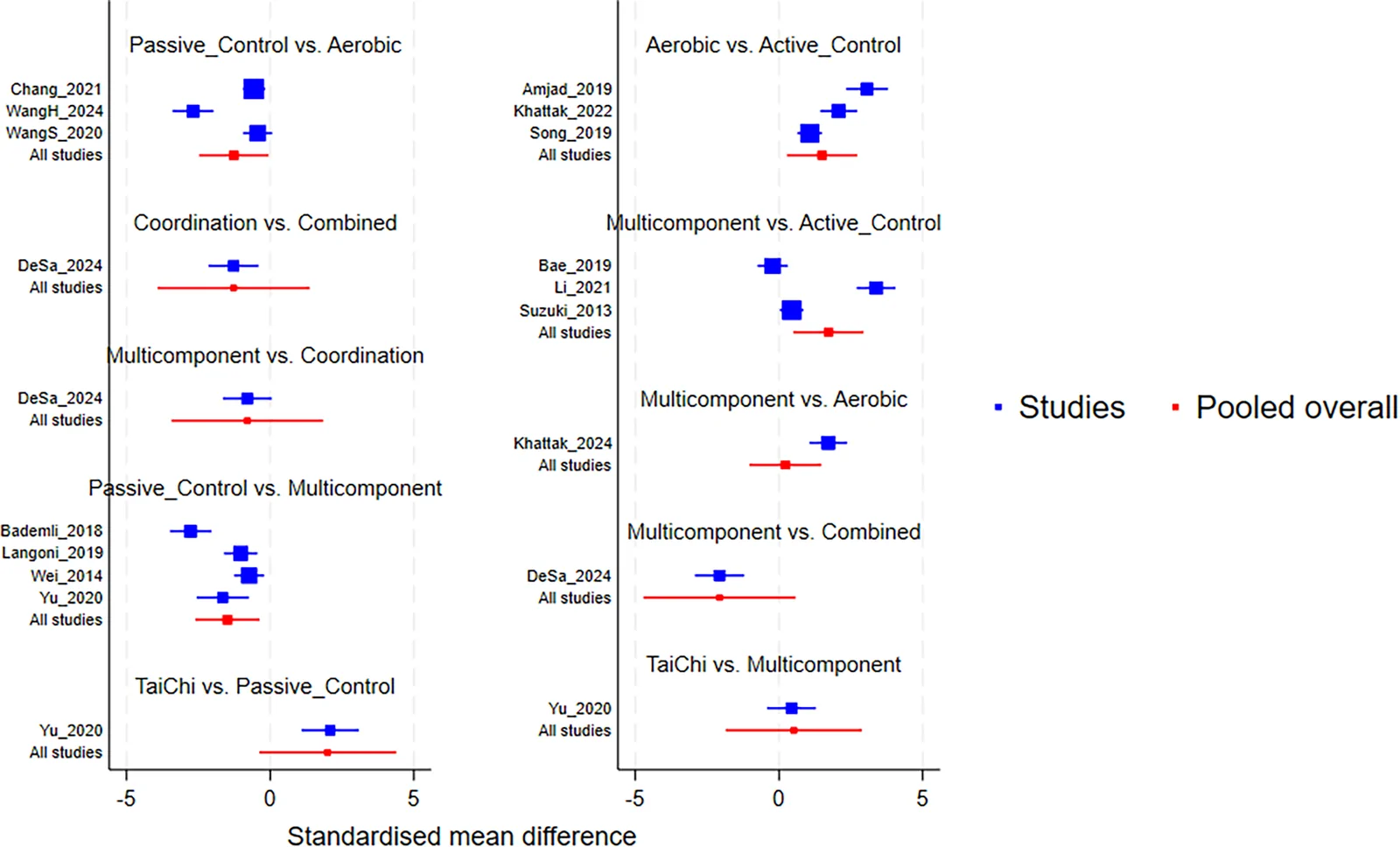
Network meta-analysis forest plot Horizontal lines indicate 95% confidence intervals.

### Ranking of exercise modalities

The intervention hierarchy was established based on SUCRA values and rank probabilities (Figure 5). Combined exercise was identified as the optimal intervention with the highest probability (68.0%) of being the best treatment for improving global cognition in MCI patients. Tai Chi ranked second with a 15.9% probability of being the best, followed by coordination training (13.3%). Passive and active controls had the highest probabilities of being the least effective interventions.

**Figure 5:**
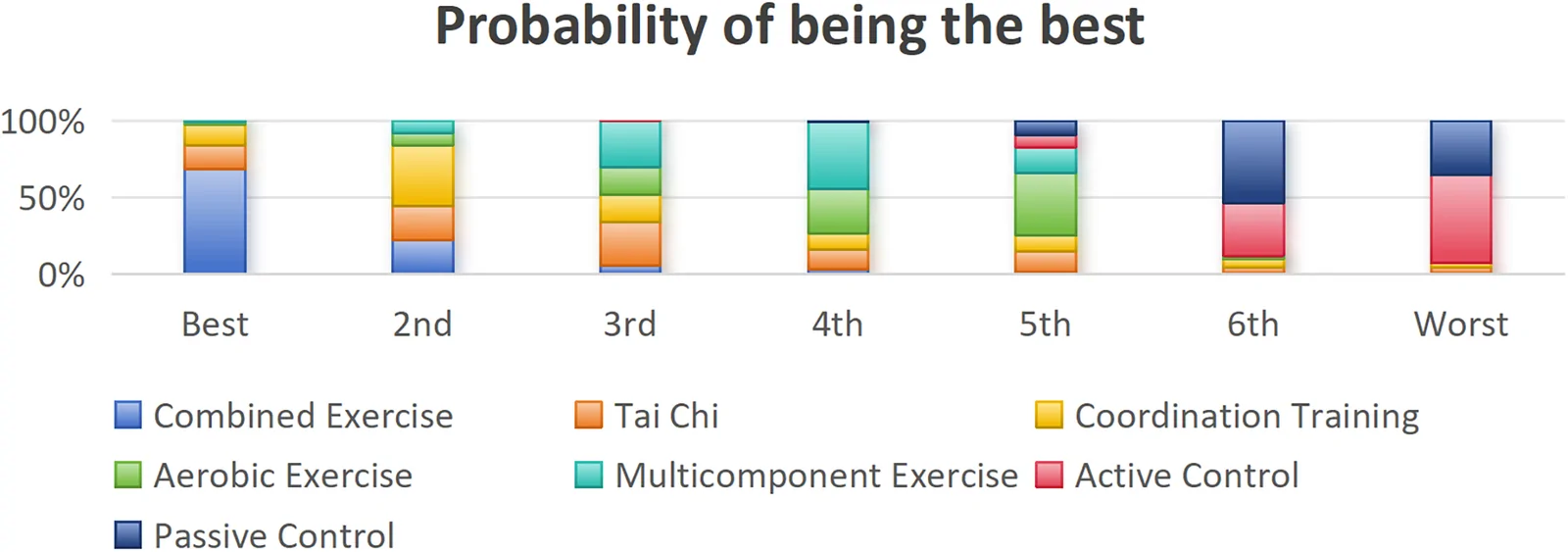
Rank probabilities of various exercise interventions

### Inconsistency and publication bias

The design-by-treatment interaction model indicated no significant global inconsistency between direct and indirect evidence in the network (*p* > 0.05). Furthermore, a comparison-adjusted funnel plot (Figure S1) was generated to assess publication bias; the symmetrical distribution of studies suggested no evidence of significant small-study effects.

## Discussion

### Principal findings

To our knowledge, this is the first NMA to simultaneously compare the efficacy of diverse specific exercise modalities on global cognitive function in older adults with MCI. Our analysis of 16 RCTs yielded a clear treatment hierarchy. Combined exercise emerged as the optimal intervention, possessing a 68.0% probability of being the most effective modality. Tai Chi and coordination training were the next most efficacious interventions. Conversely, single-mode conventional aerobic exercises showed modest benefits, while active and passive controls consistently ranked at the bottom. These findings directly address the clinical dilemma of exercise prescription, suggesting that modalities demanding high cognitive and neural engagement confer superior benefits over those requiring purely physical exertion.

### Mechanisms of combined exercise and coordination training

The overwhelming superiority of combined exercise (incorporating concurrent physical and cognitive tasks) aligns robustly with the “dual-task” and “neuroplasticity” theories. While physical exertion alone increases cerebral blood flow and upregulates the expression of brain-derived neurotrophic factor (BDNF),^29^^,^^30^ adding a simultaneous cognitive load forces the brain to allocate attention and executive resources efficiently. This synergistic stimulus has been shown to induce more profound structural and functional adaptations in neural networks, particularly in the prefrontal cortex and hippocampus, regions heavily implicated in MCI.^31^ Similarly, coordination training requires continuous motor planning, spatial orientation, and precise neuromotor control, essentially acting as an implicit form of cognitive training embedded within physical movement.

### Synergistic mechanisms of combined interventions

The superiority of combined exercise cannot be fully explained by cardiovascular improvements alone. Instead, it requires a multidimensional perspective. On a physiological level, aerobic and resistance components enhance cerebral hemodynamics and stimulate the peripheral release of neuroprotective myokines and BDNF.^32^^,^^33^ Simultaneously, the cognitive load imposed by combined interventions forces the active recruitment of executive control networks in the prefrontal cortex. This concurrent stimulation creates a synergistic environment where newly generated synapses (driven by physical activity) are immediately integrated into functional neural circuits (driven by cognitive demand), thereby maximizing cognitive reserve.

### The unique advantage of Tai Chi

Our finding that Tai Chi ranked as the second most effective intervention (15.9% probability of being the best) provides compelling quantitative evidence for traditional mind-body practices.^34^ Unlike conventional aerobic exercises (e.g., treadmill walking), which often involve repetitive and automated motor patterns, Tai Chi is a complex mind-body intervention. It necessitates sustained focused attention, sequential memorization of intricate movement routines, and deep mindful breathing. This constant requirement for conscious motor control and visuospatial memory effectively serves as a physical-cognitive dual task,^35^^,^^36^ thereby explaining its superior cognitive benefits over conventional aerobic exercise, which ranked considerably lower in our network hierarchy.

### Mind-body synergy and stress regulation

Furthermore, the high ranking of Tai Chi highlights the critical role of psychological and autonomic regulation in cognitive health. Traditional mind-body exercises engage a complex interplay of physical movement, breath control, and mindfulness. This mind-body synergy not only improves visuospatial memory and motor coordination but also modulates the autonomic nervous system, downregulating sympathetic hyperactivity and reducing systemic cortisol levels.^37^ Chronic stress and elevated cortisol are known to accelerate hippocampal atrophy in MCI.^38^ Therefore, Tai Chi may confer its superior cognitive benefits through a dual pathway: actively stimulating neural networks while simultaneously shielding the brain from stress-induced neurotoxicity.

### Limitations of the study

Despite the rigorous methodology, several limitations must be acknowledged. First, a significant limitation within the current evidence base is the inadequate reporting of specific exercise parameters (FITT principles). Many included trials failed to provide precise, quantifiable data on exercise intensity (e.g., target heart rate zones or Borg rating of perceived exertion). This lack of standardization prevents a definitive dose-response analysis. Future RCTs should strictly adhere to standardized reporting guidelines for exercise interventions, such as the CERT consensus. Second, some nodes in our network (e.g., coordination training) contained relatively few direct head-to-head comparisons, resulting in wider CIs. Larger-scale, multi-arm RCTs comparing combined training directly with Tai Chi and aerobic exercises are warranted to further solidify this treatment hierarchy. Third, there is a critical lack of reporting regarding intervention fidelity and adherence standardization across the included trials. Variability in how strictly participants adhered to the prescribed exercise regimens, and how faithfully the interventions were delivered by instructors, introduces potential confounding effects that could influence the observed cognitive outcomes.

### Conclusion

This NMA demonstrates that among older adults with MCI, combined physical-cognitive exercise is the most effective intervention for improving global cognition, followed by Tai Chi and coordination training. Interventions that demand simultaneous neural engagement and physical exertion are significantly superior to single-mode exercises or usual care. These findings offer robust, evidence-based guidance for clinicians and therapists to prescribe precise, dual-component exercise regimens to mitigate cognitive decline in high-risk populations.

## Resource availability

### Lead contact

Further information and requests for resources should be directed to and will be fulfilled by the lead contact, Yin Wu (wuyin@suda.edu.cn).

### Materials availability

This study did not generate new unique reagents or biological materials. All data analyzed were extracted from previously published randomized controlled trials listed in the reference list.

### Data and code availability

All raw outcome data supporting the findings are available in the original published articles included in this systematic review. The extracted dataset and Stata code for the network meta-analysis are available from the lead contact upon reasonable request. The study protocol was prospectively registered in the PROSPERO database (Registration number: CRD420251026910).

## Acknowledgments

The authors would like to express sincere gratitude to Can Wang for her invaluable assistance in independent literature screening and preliminary data extraction, which ensured the rigorous double-blind procedure required by PRISMA guidelines.

## Author contributions

Conceptualization: Yixuan Wu and Yin Wu; methodology: Yixuan Wu and Yin Wu; formal analysis: Yixuan Wu; data curation: Yixuan Wu; writing – original draft: Yixuan Wu; writing – review and editing: Yin Wu; supervision: Yin Wu

## Declaration of interests

The authors declare no competing interests.

## STAR★Methods

### Key resources table

**Table:** 

REAGENT or RESOURCE	SOURCE	IDENTIFIER
**Deposited data**		

International prospective register of systematic reviews	PROSPERO	Registration No. CRD420251026910; https://www.crd.york.ac.uk/PROSPERO/
Studies for meta-analysis	PubMed, Embase, Web of Science, Cochrane Library	The included studies are referenced in the reference list and Table 1.

**Software and algorithms**		

Stata (version 18.0)	StataCorp LLC	https://www.stata.com/
EndNote 21	Clarivate Analytics	https://endnote.com/

### Method details

#### Study design and registration

This systematic review and network meta-analysis was conducted in strict accordance with the Preferred Reporting Items for Systematic Reviews and Meta-Analyses (PRISMA) guidelines,^39^ as well as the PRISMA extension statement for Network Meta-Analysis (PRISMA-NMA).^40^ The study protocol was prospectively registered in the PROSPERO database (Registration number: CRD420251026910).

#### Literature search

A comprehensive literature search was systematically performed across four electronic databases: PubMed, Embase, Web of Science and Cochrane Library, from inception to April 2026. The search strategy combined Medical Subject Headings (MeSH) and free-text terms related to “mild cognitive impairment”, “exercise”, “physical activity”, and “randomized controlled trial”. Furthermore, reference lists of relevant systematic reviews and included trials were manually screened to identify any additional eligible studies. The detailed search syntax is provided in Table S1.

#### Eligibility criteria

Studies were selected based on the PICOS (Population, Intervention, Comparison, Outcome, Study design) framework: (1) **Population:** Older adults (aged ≥60 years) diagnosed with mild cognitive impairment (MCI) according to recognized clinical criteria. (2) **Intervention:** Any structured physical exercise program, which was further classified into specific modalities for network comparison (e.g., aerobic exercise, multicomponent exercise, coordination training, Tai Chi, and combined physical-cognitive training). (3) **Comparison:** Active control (e.g., health education, stretching) or passive control (e.g., usual care, no intervention). (4) **Outcome:** Global cognitive function, assessed by validated standardized tools, such as the Montreal Cognitive Assessment (MoCA) or Mini-Mental State Examination (MMSE). (5) **Study design:** Randomized controlled trials (RCTs). We excluded observational studies, animal studies, non-English publications, and trials lacking extractable quantitative data.

#### Study selection and data extraction

Two independent reviewers screened the titles and abstracts of the retrieved records. Full texts of potentially eligible articles were subsequently assessed against the inclusion criteria. Discrepancies were resolved through discussion or consultation with a third reviewer. Data extraction was independently performed using a standardized, pre-piloted spreadsheet. Extracted data included study characteristics (first author, publication year, country), participant demographics (sample size, sex, mean age), intervention details (type, frequency, duration), and the means and standard deviations (SDs) of post-intervention cognitive scores.

#### Risk of bias assessment

The methodological quality of the included RCTs was evaluated using the revised Cochrane risk-of-bias tool for randomized trials (RoB 2.0).^41^ Two investigators independently assessed five domains of bias: (1) bias arising from the randomization process, (2) bias due to deviations from intended interventions, (3) bias due to missing outcome data, (4) bias in measurement of the outcome, and (5) bias in selection of the reported result. Each domain, as well as the overall risk of bias, was graded as “low risk,” “some concerns,” or “high risk.”

### Quantification and statistical analysis

All statistical analyses were performed using Stata software (version Stata 18.0, Stata Corp, TX, USA) using the network package. We conducted a frequentist network meta-analysis to simultaneously synthesize direct and indirect evidence. Since different cognitive scales MoCA and MMSE were utilized across studies, treatment effects were estimated using standardized mean differences (SMDs) with 95% confidence intervals (CIs). First, a network plot was generated to visualize the geometry of the evidence network, where node sizes reflect the total sample size for each intervention and edge thicknesses represent the number of direct comparisons. Second, we assessed the global consistency of the network (the agreement between direct and indirect evidence) using the design-by-treatment interaction model. A random-effects model was applied if significant heterogeneity or inconsistency was observed; otherwise, a common-effect model was adopted. Finally, to establish a clinically meaningful hierarchy of the exercise modalities, we calculated the Surface Under the Cumulative Ranking curve (SUCRA) and the rank probabilities for each intervention. SUCRA values range from 0 to 100%, with higher values indicating a higher probability of an intervention being the most effective treatment for improving global cognition. Additionally, the certainty of evidence for network estimates was evaluated using the Confidence in Network Meta-Analysis (CINeMA) framework).

## Supplemental information

Document S1. Table S1

Data S1. PRISMA_NMA_checklist.pdf
