# Supplementary material for: Comparative efficacy of exercise modalities for mild cognitive impairment in older adults: A network meta-analysis
Source: iScience. 2026 Oct 7;29(11):117561. doi: 10.1016/j.isci.2026.117561 (PMC13676722; doi:10.1016/j.isci.2026.117561)
Supplement: Supplementary file 1 [file mmc1.pdf]

iScience, Volume *29*

Supplemental information

Comparative efficacy of exercise modalities for mild cognitive impairment in older adults: A network meta-analysis

Yixuan Wu and Yin Wu

Table S1. Search strategy for electronic databases

Database	Search Strategy
**PubMed**	("Cognitive Dysfunction"[Mesh] OR "mild cognitive impairment"[Title/Abstract] OR "MCI"[Title/Abstract] OR "mild neurocognitive disorder"[Title/Abstract] OR "cognitive decline"[Title/Abstract]) AND ("Exercise"[Mesh] OR "Exercise Therapy"[Mesh] OR "Resistance Training"[Mesh] OR "Tai Ji"[Mesh] OR "Yoga"[Mesh] OR "conventional exercise"[Title/Abstract] OR "aerobic exercise"[Title/Abstract] OR "resistance training"[Title/Abstract] OR "strength training"[Title/Abstract] OR "physical activity"[Title/Abstract] OR "mind-body exercise"[Title/Abstract] OR "Tai Chi"[Title/Abstract] OR "Taiji"[Title/Abstract] OR "Yoga"[Title/Abstract] OR "Qigong"[Title/Abstract] OR "Baduanjin"[Title/Abstract] OR "dance"[Title/Abstract]) AND ("Cognition"[Mesh] OR "cognitive function"[Title/Abstract] OR "global cognition"[Title/Abstract] OR "executive function"[Title/Abstract] OR "memory"[Title/Abstract] OR "Mini-Mental State Examination"[Title/Abstract] OR "MMSE"[Title/Abstract] OR "Montreal Cognitive Assessment"[Title/Abstract] OR "MoCA"[Title/Abstract])
**Web of Science**	TS=("mild cognitive impairment" OR "MCI" OR "mild neurocognitive disorder" OR "cognitive decline") AND TS=("exercise" OR "conventional exercise" OR "aerobic exercise" OR "resistance training" OR "strength training" OR "physical activity" OR "mind-body exercise" OR "Tai Chi" OR "Taiji" OR "Yoga" OR "Qigong" OR "Baduanjin" OR "dance") AND TS=("cognition" OR "cognitive function" OR "global cognition" OR "executive function" OR "memory" OR "Mini-Mental State Examination" OR "MMSE" OR "Montreal Cognitive Assessment" OR "MoCA")
**Embase**	('mild cognitive impairment'/exp OR 'mild cognitive impairment':ti,ab,kw OR 'MCI':ti,ab,kw OR 'mild neurocognitive disorder':ti,ab,kw OR 'cognitive decline':ti,ab,kw) AND ('exercise'/exp OR 'resistance training'/exp OR 'tai chi'/exp OR 'yoga'/exp OR 'conventional exercise':ti,ab,kw OR 'aerobic exercise':ti,ab,kw OR 'resistance training':ti,ab,kw OR 'strength training':ti,ab,kw OR 'physical activity':ti,ab,kw OR 'mind-body exercise':ti,ab,kw OR 'tai chi':ti,ab,kw OR 'taiji':ti,ab,kw OR 'yoga':ti,ab,kw OR 'qigong':ti,ab,kw OR 'baduanjin':ti,ab,kw OR 'dance':ti,ab,kw) AND ('cognition'/exp OR 'cognitive function':ti,ab,kw OR 'global cognition':ti,ab,kw OR 'executive function':ti,ab,kw OR 'memory':ti,ab,kw OR 'mini-mental state examination':ti,ab,kw OR 'mmse':ti,ab,kw OR 'montreal cognitive assessment':ti,ab,kw OR 'moca':ti,ab,kw) AND [english]/lim AND ('randomized controlled trial'/exp OR 'randomized controlled trial (topic)'/exp)
**Cochrane Library**	("mild cognitive impairment" OR "MCI" OR "mild neurocognitive disorder" OR "cognitive decline"):ti,ab,kw AND ("exercise" OR "conventional exercise" OR "aerobic exercise" OR "resistance training" OR "strength training" OR "physical activity" OR "mind-body exercise" OR "Tai Chi" OR "Taiji" OR "Yoga" OR "Qigong" OR "Baduanjin" OR "dance"):ti,ab,kw AND ("cognition" OR "cognitive function" OR "global cognition" OR "executive function" OR "memory" OR "Mini-Mental State Examination" OR "MMSE" OR "Montreal Cognitive Assessment" OR "MoCA"):ti,ab,kw

Table S2. Confidence in Network Meta-Analysis (CINeMA) assessment for the primary outcome (Global Cognition vs. Active Control)

Comparison (Intervention vs. Active Control)	Within-study bias	Reporting bias	Indirectness	Imprecision	Heterogeneity	Incoherence	Overall Confidence
**Combined Exercise**	Major concerns	No concerns	No concerns	Some concerns	Some concerns	No concerns	**Low**
**Tai-Chi**	Major concerns	No concerns	No concerns	Some concerns	Some concerns	No concerns	**Low**
**Multicomponent Exercise**	Major concerns	No concerns	No concerns	No concerns	Some concerns	No concerns	**Moderate**
**Aerobic Exercise**	Major concerns	No concerns	No concerns	No concerns	Some concerns	No concerns	**Moderate**
**Coordination Training**	Major concerns	No concerns	No concerns	Major concerns	No concerns	No concerns	**Very Low**

Figure S1. Comparison-adjusted funnel plot for the network meta-analysis of exercise modalities on global cognition

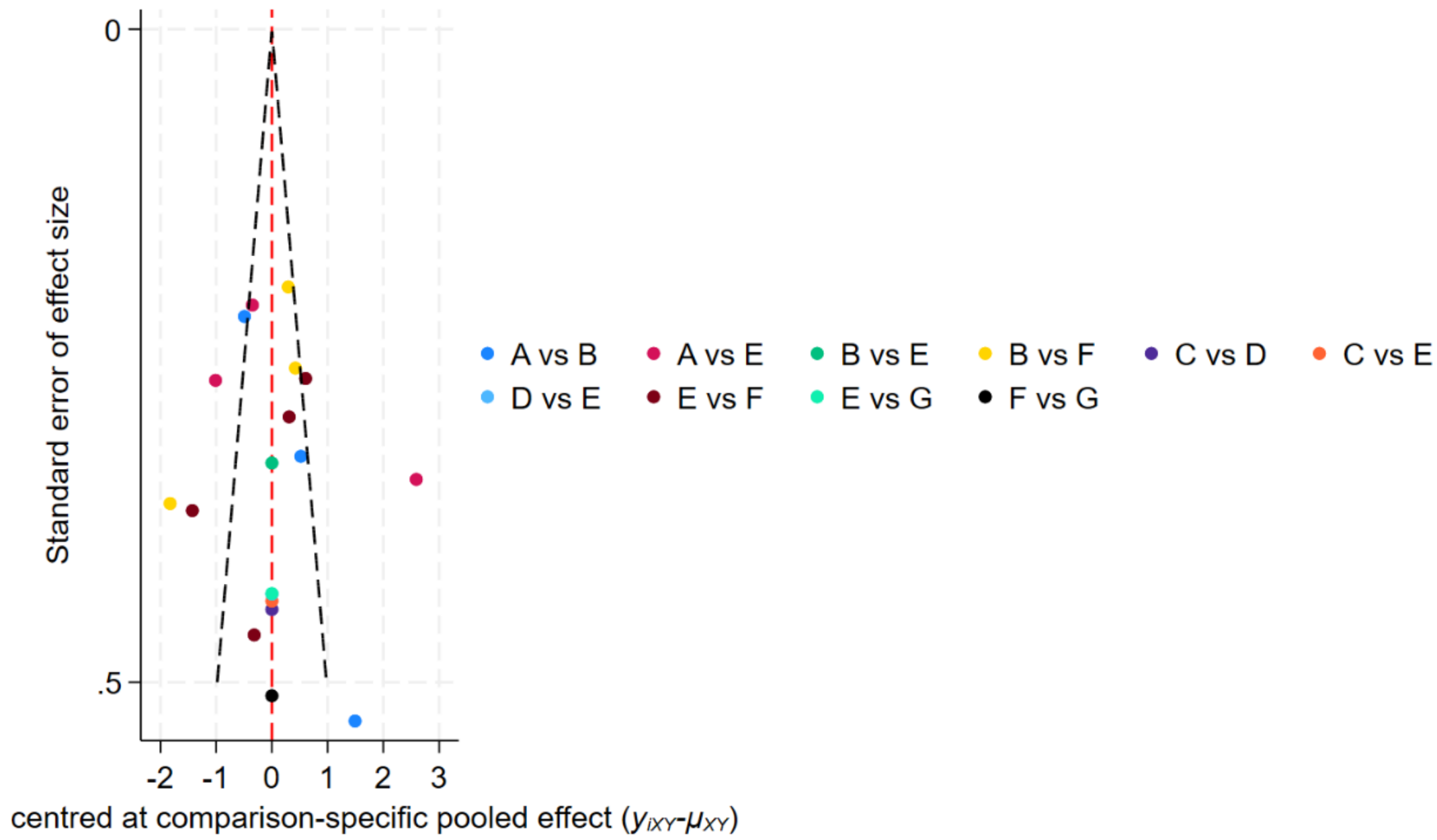

Each dot represents an individual study comparison, and different colors indicate different treatment comparisons. The horizontal axis represents the effect size centered at the comparison-specific pooled effect, and the vertical axis represents the standard error of the effect size. The comparison-adjusted funnel plot was used to assess potential small-study effects. Treatment codes are defined as follows: A, Active Control; B, Aerobic Exercise; C, Combined Exercise; D, Coordination Training; E, Multicomponent Exercise; F, Passive Control; G, Tai Chi.
